# Pre-emptive versus empirical antifungal therapy in patients with febrile neutropenia with acute leukaemia: a GIMEMA study

**DOI:** 10.1093/jacamr/dlag120

**Published:** 2026-06-26

**Authors:** Alessandra Micozzi, Giuseppe Gentile, Marco Picardi, Clara Minotti, Stelvio Ballanti, Mario Delia, Federico Simonetti, Bruno Martino, Antonella Ferrari, Francesco Lanza, Angelo M Carella, Prassede Salutari, Lorella M A Melillo, Monica Bocchia, Mario Luppi, Mara Merluzzi, Giampaolo Bucaneve, Gruppo Italiano Malattie Ematologiche dell’Adulto (GIMEMA) Infection Program Working Party, Fabrizio Pane, Maria Paola Martelli, Pellegrino Musto, Enrico Capochiani, Massimo Martino, Monica Piedimonte, Eliana Zuffa, Nicola Cascavilla, Mauro Di Ianni

**Affiliations:** Ematologia, Dipartimento di Medicina Traslazionale e di Precisione, Sapienza Università di Roma, Roma, Italy; Ematologia, Dipartimento di Medicina Traslazionale e di Precisione, Sapienza Università di Roma, Roma, Italy; Dipartimento di Medicina Clinica e Chirurgia, Università Federico II, Napoli, Italy; Dipartimento di Ematologia Oncologia e Dermatologia, Azienda Policlinico Umberto I di Roma, Roma, Italy; Ematologia e TMO, Azienda Ospedaliera di Perugia, Ospedale S. M. Della Misericordia, Perugia, Italy; Ematologia e Trapianto di Cellule Staminali, Azienda Ospedaliero-Universitaria-Consorziale (AOUC) Policlinico di Bari, Bari, Italy; Azienda USL Toscana Nord Ovest, UOC Ematologia Aziendale Ospedale Versilia, Lido di Camaiore, Italy; Dipartimento di Emato-Oncologia e Radioterapia, Grande Ospedale Metropolitano Bianchi-Melacrino-Morelli, Reggio Calabria, Italy; Ematologia, Azienda Ospedaliera Universitaria Sant’Andrea, Dipartimento di Medicina Clinica e Molecolare, Sapienza Università di Roma, Roma, Italy; Dipartimento Universitario DIMEC, Alma Mater Università di Bologna, Ematologia, Ospedale di Ravenna, Ravenna, Italy; Ematologia e Centro Trapianti, Fondazione Casa Sollievo della Sofferenza IRCCS, San Giovanni Rotondo, Italy; Ematologia, Dipartimento Oncologico Ematologico, Ospedale di Pescara, Pescara, Italy; Ematologia, Policlinico di Foggia, Foggia, Italy; Ematologia, Università di Siena, Azienda Ospedaliero-Universitaria Senese, Siena, Italy; Ematologia, Policlinico di Modena, Modena, Italy; Ematologia ed Immunologia Clinica, Dipartimento di Medicina e Chirurgia, Università degli Studi di Perugia, Perugia, Italy; Medicina Interna e Scienze Oncologiche, Azienda Ospedaliera di Perugia—Centro Regionale di Farmacovigilanza, Regione Umbria, Perugia, Italy

## Abstract

**Objectives:**

To compare the efficacy of pre-emptive antifungal therapy (PAT) and empirical antifungal therapy (EAT) in patients with febrile neutropenic acute leukaemia (AL) with/without anti-mold prophylaxis.

**Methods:**

We included 92 patients with AL and neutropenia with fever unresponsive to empiric antibiotics, stratified by posaconazole prophylaxis to receive EAT (*n* = 40) or PAT (*n* = 52). In the PAT group, a standardized diagnostic workup for the detection of invasive fungal infection (IFI) was performed. The study endpoints were antifungal therapy initiation, IFI documentation, safety, and cost.

**Results:**

Posaconazole prophylaxis was equally distributed among the patients (overall 55.4%). Antifungal therapy was started in 58% of the patients in the PAT group versus 100% in the EAT group (*P* < 0.01; 95% CI: 0.42 [0.28–0.55]). In the EAT and PAT groups, overall proven/probable IFI rates were 2% and 17%, respectively (*P* = 0.02; 95%CI: −0.14 [−0.26 to −0.03]) and 4% and 10% in patients with posaconazole prophylaxis, respectively (*P* = 0.3; 95% CI: −0.06 [−0.20–0.07]). At hospital discharge, mortality was 19% in the EAT and 5% in the PAT groups (*P* = 0.02; 95% CI: −0.14 [−0.26 to −0.03]), unrelated to the use of posaconazole prophylaxis, and no IFI-related death was observed. The mean costs per patient were €7681 and €3344 in the EAT and PAT groups, respectively (*P* = 0.003; 95% CI: €4337 [€1814–6860]). Adverse reactions to antifungals were similar between the groups.

**Conclusions:**

In patients with persistently febrile neutropenic AL, PAT was safe and effective and reduced the use and costs of antifungals, irrespective of anti-mold prophylaxis, compared with empirical treatment.

## Introduction

In patients with persistent febrile neutropenia with acute leukaemia (AL), the pre-emptive approach for the treatment of invasive fungal infections (IFIs) (pre-emptive antifungal therapy [PAT]), a diagnosis-driven strategy aimed at avoiding or more appropriately targeting antifungal treatment to be initiated in the presence of clinical findings, mycological evidence, and/or imaging studies, is associated with lower antifungal exposure without an increase in IFI-related mortality or overall mortality and promising results in terms of clinical outcomes and cost effectiveness.^1–5^ However, there is no consensus on the optimal design of the pre-emptive strategy, so that even today the empirical antifungal therapy (EAT) has remained the recommended standard in this setting.^6,7^

In this multicentre, randomized clinical trial, we compared the efficacy of PAT with EAT in persistently febrile neutropenic patients with AL undergoing intensive chemotherapy, including and stratifying patients on oral anti-mold prophylaxis.

## Materials and methods

Prospective, multicentre, randomized trial conducted between April 2010 and January 2015 at 13 haematological centres of the Gruppo Italiano Malattie EMatologiche dell’Adulto (GIMEMA) in Italy.

Consecutive adult patients with AL undergoing intensive chemotherapy were eligible if they had fever unresponsive to at least 96 h of adequate empiric antibiotic treatment or recurrent after temporary improvement, chemotherapy-induced neutropenia for at least 10 days, and a negative initial work-up to exclude bacterial infections. All oral antifungal prophylaxis was allowed. (Annex 1: Study Protocol, point 3.2: Inclusion Exclusion Criteria).

The primary objective of this study was to compare the efficacy (number of patients undergoing antifungal therapy) of PAT with EAT strategies. The secondary objectives were to compare the number of documented IFIs, safety, costs, the survival rates, clinical outcomes, and use of other healthcare resources of PAT with those of EAT. The hypothesis was that PAT is related to a lower use of antifungals, lower drug-induced toxicity and costs, and more efficient IFI documentation, with at least similar outcomes.

After randomization, patients assigned to receive EAT started antifungal treatment immediately. Patients assigned to receive PAT underwent a standardized diagnostic workup (SDWU) (Study Protocol), starting the antifungal treatment only if a proven, probable, or possible IFI was documented^8^ or if they fell into the ‘febrile neutropenia plus one diagnostic factor’ category (FN + 1 category) (Study Protocol).

The indicated antifungal treatments in the EAT group were liposomal amphotericin B (3 mg/kg per day), amphotericin B lipid complex (5 mg/kg per day), or caspofungin (70 mg loading dose, then 50 mg per day) for a minimum duration of 2 weeks. In the PAT group, antifungal treatment could be targeted according to the diagnosis of IFIs (polyenes, azoles, and echinocandins).

Randomization, sample size estimation, and statistical analysis are described in the Study Protocol. Patients were stratified by centre and the use of posaconazole prophylaxis.

## Results

A total of 101 patients underwent randomization (intention-to-treat [ITT] population); 42 were assigned to receive EAT and 59 received PAT. The per-protocol analysis population included 92 patients (Figure S1, available as Supplementary data at *JAC-AMR* Online). The characteristics of the patients were similar between the groups (Table S1).

Antifungal therapy was started in 100% (40/40) and 58% (30/52) of patients randomized to the EAT and PAT group, respectively (*P* < 0.01; 95% CI: 0.42[0.28–0.55]). In the PAT group, the reason for the initiation of antifungal therapy was the documentation of a proven, probable, or possible IFI in 20 patients (38%) and the achievement of the FN + 1 category in 10 (19%) (Table 1).

**Table 1.** Antifungal therapy, diagnostic tools, and costs (per-protocol population)

Characteristics	Empiric *n* = 40	Pre-emptive *n* = 52	CI (95%) between difference	*P*-value
Antifungal therapy	40/40 (100%)	30/52 (58%)	0.42 (0.28 to 0.55)	<0.01
Reasons for antifungal treatment start:				
proven, probable, possible IFI	—	20/52 (39%)		
FN + 1^a^	—	10/52 (19%)		
Use of antifungal therapy in patients without proven or probable IFI at final evaluation	39/40 (97%)	9/30 (30%)	0.27 (−0.44 to −0.10)	<0.01
Initial antifungal therapy (treated patients):				
Polyenes	32/40 (80%)	16/30 (53%)	0.27 [0.04 to 0.48]	0.02
Amphotericin B lipid complex	6	3		
Liposomal amphotericin B	26	13		
Azoles	2/40 (5%)	11/30 (37%)	−0.32 [−0.50 to −0.13]	<0.01
(Voriconazole)				
Echinocandins	6/40 (15%)	3/30 (10%)	0.05 [−0.10 to 0.20]	0.40
Caspofungin	5	3		
Anidulafungin	1	—		
Type of antifungal therapy including subsequent modifications (treated patients):				
Polyenes	34/40 (85%)	18/30 (60%)	0.25 [0.04 to 0.45]	0.03
Liposomal amphotericin B	27	14		
Amphotericin B lipid complex	7	4		
Azoles	5/40 (13%)	15/30 (50%)	−0.37 [−0.58 to −0.17]	<0.01
Voriconazole	5	13		
Others	—	2		
Echinocandins	10/40 (25%)	5/30 (17%)	0.08 [−0.10 to −0.27]	0.2
Caspofungin	8	4		
Others	2	1		
Duration of antifungal therapy:				
All randomized patients, mean days–(range)	17 (4–95)	12 (0–60)	5 (−1.63 to 11.6)	0.21
Only patients who underwent antifungal therapy	17 (4–95)	22 (3–60)	−5 (−12.6 to 2.62)	0.6
Performed diagnostic tests:				
Nr. of patients with thoracic CT scan	35/40 (87%)	52/52 (100%)	−0,12 [−022 to −0.02]	0.03
Nr. of patients with maxillary CT scan	4/40 (10%)	8/52 (15%)	−0.05 [−0.18 to 0.08]	0.3
Nr. of patients with galactomannan test	32/40 (80%)	52/52 (100%)	−0.20 [−0.32 to −0.07]	<0.01
Nr. of patients with nasal swab at the start of antifungal therapy	21/40 (52%)	52/52 (100%)	−47.5 [−0.63 to −0.32]	<0.01
Costs associated with the assigned strategy:				
Mean cost of antifungal therapy—€ (range):*^b^*				
All randomized patients	7470	3091	4379	<0.01
	(1.531–52 160)	(0–14 996)	(1861 to 6897)	
Only patients who underwent antifungal therapy	7470	4811	2033	0.16
	(1531–52 160)	(1488–14 996)	(−1146 to 5212)	
Mean cost of diagnostic tests Euro (range)	211	253	−42 [−102 to 17,8]	0.15
	(7–800)	(143–831)		
Total cost Euro (range) of all randomized patients	7681	3344	4337 [1814 to 6860]	<0.01
	(1662 to 52 339)	(143–15 151)		

CT, computed tomography; IFI, invasive fungal infection.

^a^FN + 1:‘febrile neutropenia plus one diagnostic factor’ additional category defined in the study protocol (Annex 1) including diagnostic criteria other than those listed as ‘major’ by the EORTC/MSG (Reference: 8).

^b^Calculations are performed using the Italian National Health Service costs of drugs and diagnostic tools (February 2020 prices). The cost of the therapy is estimated by considering the intravenous formulation of the drug for patients weighing 70 kg. (Annex 2).

The antifungal drugs used for the initial treatment were amphotericin B (liposomal or lipid complex formulation) in 80% and 53% (*P* = 0.02; 95% CI: 0.27 [0.04–0.48]), voriconazole in 5% and 37% (*P* < 0.01; 95% CI: −0.32[−0.50 to −0.13]) in the EAT and PAT groups, respectively (Table 1).

The mean duration of antifungal treatment was similar between the two groups when comparing all randomized patients (independent of antifungal treatment administration) and only patients who received antifungal treatment (Table 1).

A multivariate analysis identified the pre-emptive strategy, but not age, sex, length of neutropenia, or posaconazole prophylaxis, as the only factor capable of influencing the initiation of antifungal treatment (Table S2).

Overall, 10 proven or probable IFIs were documented in 92 patients (IFI incidence: 10.8%); one case (2%) among the 40 patients in the EAT group and nine (17%) among the 52 patients in the PAT group (*P* = 0.02; 95% CI: −0.14[−0.26 to −0,03]) (Table 2, Table S3).

**Table 2.** Fungal infections, mortality, and safety

Characteristics	Empiric	Pre-emptive	CI (95%) between difference	*P*-value
**Per protocol analysis**				
	*n* = 40	*n* = 52		
Fungal infections at the end of the episode	3/40 (7%)	18/52 (35%)	−0.28 (−0.42 to −0.11)	<0.01
Proven	0	2		
Probable	1	7		
Possible	2	9		
No fungal infections		2		
Proven and probable IFIs:				
Overall	1/40 (2%)	9/52 (17%)	−0.14 (−0.26 to −0.03)	0.02
Patients receiving antifungal therapy	1/40 (2%)	9/30 (30%)	−0.27 (−0.44 to −0.10)	< 0.01
Patients under posaconazole prophylaxis	1/23 (4%)	3/28 (10%)	−0.06 (−0.20 to 0.07)	
Patients under other or no prophylaxis	0/17 (0%)	6/24 (25%)	−0.20 (−0.40 to −0.01)	0.05
Site of infection:				
Lung	1/40 (2%)	7/52 (13%)	0.11 (−0.21 to −0.004)	0.06
CVC/fungaemia	—	2/52 (4%)		
**ITT analysis:**				
	*n* = 42	*n* = 59		
Mortality				
Overall	8/42 (19%)	3/59 (5%)	0.14 (0.008 to 0.26)	0.02
In patients with posaconazole prophylaxis	4/42 (9%)	2/59 (3%)	0.06 (−0.03 to 0.16)	0.2
In patients with other or no prophylaxis	4/42 (9.5%)	1/59 (2%)	0.07 (−0.016 to 0.17)	0.1
Cause of death :				
Infection	1	2		
Bacterial	1	1		
Viral	—	1		
Underlying disease progression	**2**	**—**		
Other causes	**5*^a^***	**1*^b^***		
Adverse drug reactions (overall):	4/42 (9%)	3/59 (5%)	0.04 (−0.06 to 0.14)	0.3
Renal failure	0	1		
Fever or skin rash	4	2		

CVC, central venous catheter; IFI, invasive fungal infection.

^a^Pulmonary embolism (*n* = 1), hepatic failure (*n* = 1), myocardial infarction (*n* = 1), and cerebral bleeding (*n* = 2).

^b^Interstitial pneumopathy.

By evaluating only patients who received antifungal treatment, proven or probable IFIs were documented in 3% (1/40) and 30% (9/30) in EAT and PAT group, respectively (45% [9/20]; *P* = 0.001; 95% CI: −0.27[−0.44 to −0.10]) (Table 2).

In the PAT group, no proven or probable IFIs were documented among the 10 patients in whom antifungal treatment was initiated for FN + 1, and a possible IFI was found in only one case (Table S4). Excluding these patients, proven or probable IFIs were documented in 45% of patients who received antifungal treatment.

Antifungal treatment was administered without a final diagnosis of proven or probable IFI in 97% and 30% of patients in the EAT and PAT groups, respectively (*P* = 0.003; 95% CI: 0.27[−0.44 to −0.10]) (Table 1).

Among the 51 patients undergoing posaconazole prophylaxis, one probable IFI was found in 23 patients in the EAT group (4%) and three proven or probable IFIs in 28 patients in the PAT group (10%) (*P* = 0.3; 95% CI: −0.06[−0.20–0.07]). Among patients undergoing other or no prophylaxis, no proven or probable IFIs were found in 17 patients in the EAT group compared to six of the 24 patients in the PAT group (25%) (*P* = 0.05; 95%CI: −0.20[−0.40 to −0.07]) (Table 2, Table S5).

In the ITT population (*n* = 101), there were 11 deaths (10.9%) during the observation period (discharge from the hospital), 8 (19%) and 3 (5%) in the EAT and PAT groups, respectively (*P* = 0.02; 95% CI: −0.14[−0.26 to −0.03]), with no differences between patients with/without posaconazole prophylaxis (Table 2). No IFI-related death was observed.

All adverse drug reactions occurred in patients receiving liposomal or lipid amphotericin B. Similarly, in the two study groups (9.5% and 8.6% in the ETA and PTA groups, respectively), the adverse drug reactions were mild in all but one case of renal failure (Table 2).

The types and numbers of fungal infection diagnostic tests performed in the two study groups are shown in Table 1. According to the SDWU, patients in the PAT group underwent more diagnostic examinations (CT scan, GM test and nasal swab) than those in the EAT group. No patient underwent bronco-alveolar lavage (not included in the SDWU).

All patients in the PAT group had at least one thoracic computed tomography scan, one serum galactomannan test, and one nasal swab for microbiological examination performed, in comparison with the 87% (*P* = 0.03; 95% CI: −0.12[−0.22 to −0.02]), 80% (*P* < 0.01; 95% CI: −0.20[−0.32 to −0.07]), and 52% (*P* < 0.0001; 95% CI: −47.5[−0.63 to −032]), respectively, performed in patients in the EAT group.

The cost of a single drug, microbiological test, and imaging test are reported in euros (Annex 2). A significant difference between the two groups was observed in the mean cost of antifungal treatment: €7470 and €3091 (*P* = 0.003; 95% CI: €4379[€1861–6897]) for all patients, €7470 and €4811 for patients who underwent antifungal treatment in the EAT and PAT groups, respectively. The mean cost of the diagnostic test for the randomized patients was similar (€211 and €253 in the EAT and PAT groups, respectively). Overall, the total mean cost of the assigned strategy per patient resulted €7681 in the EAT group and €3344 in the PAT group (*P* = 0.003; 95% CI: €4337[€1814–6860]) (Table 1).

## Discussion

We found that PAT significantly reduced the number of patients undergoing antifungal therapy compared to those receiving EAT, irrespective of the use of anti-mold prophylaxis. No cases of IFI-related death were observed. A lower death rate was found in favour of the PAT group, but this finding should be interpreted with caution because the trial sample size was not powered to detect differences in mortality.

Our results are in agreement with those of meta-analyses published in 2015^9^ and 2022,^10^ confirmed in the European Organization for Research and Treatment Cancer (EORTC) study,^11^ which showed that PAT was safe for high-risk patients with neutropenia undergoing fluconazole prophylaxis, thereby reducing the administration of antifungals without excess mortality or IFIs.

In our study, the incidences of probable and proven IFIs were significantly higher in the PAT group, according to the results of meta-analyses^9,10^ but in contrast to recent randomized trials in paediatric and adult patients mostly affected by haematological malignancies.^12,13^ Our results did not suggest that FN + 1 category is specific to IFI.

The small size of the trial and the long delay between study completion and submission, explained primarily by the loss of one-third of the centres that had initially agreed to the study, the temporary loss of funding, and the COVID pandemic, are limitations of the study that reduce the strength of the results and prevent firm conclusions.

In conclusion, our study provides further evidence that PAT is safe, less costly, and more efficient for the documentation of IFIs and addresses relevant questions in antifungal stewardship in high-risk patients with AL with persistent febrile neutropenia: the diagnostic-driven approach appears to favour the appropriate prescription of antifungal drugs, and the initiation of antifungal therapy or the pre-emptive strategy seem to be unaffected by the use of anti-mold prophylaxis.

## Supplementary Material

dlag120_Supplementary_Data

## Transparency declarations

We declare no competing interests. A.M. reports support for attending meetings and travel from Gilead Sciences and Pfizer. G.G. reports payment or honoraria for lectures, presentation, speaker bureaus, manuscript writing or educational events from Gilead Sciences and Pfizer and support for attending meeting and travel from Gilead Sciences. F.L. reports participation on Data Safety Monitoring Board or Advisory Board from Amgen—Golden Gate International Trial. PS reports support for attending meetings and travel from Sanofi and Novartis. L.M.A.M. reports support for attending meetings and travel from Incyte and Novartis. M.L. reports payment or honoraria for lectures, presentation, speaker bureaus, manuscript writing or educational events from Abbvie, Grifols, Incyte, Istituto Gentili, Roche, Jazz Pharma, Novartis, Astrazeneca.

## Supplementary data

Figure S1 and Tables S1 to S5 are available as Supplementary data at *JAC-AMR* Online.
