# Supplementary material for: Pre-emptive versus empirical antifungal therapy in patients with febrile neutropenia with acute leukaemia: a GIMEMA study
Source: JAC Antimicrob Resist. 2026 Jun 26;8(3):dlag120. doi: 10.1093/jacamr/dlag120 (PMC13308701; doi:10.1093/jacamr/dlag120)
Supplement: dlag120_Supplementary_Data [file dlag120_supplementary_data.zip › Annex 1 Study PROTOCOL Versione 2. Orginale AIFA. 26-03-2009 inglese.pdf]

GIMEMA Infection Program
Gruppo Italiano Malattie Ematologiche dell'Adulto

PROTOCOLLO
Versione 2. 26/03/2009

STUDIO PROSPETTICO, APERTO, RANDOMIZZATO, MULTICENTRICO DI CONFRONTO TRA DUE APPROCCI TERAPEUTICI PER IL TRATTAMENTO DELLE INFEZIONI FUNGINE INVASIVE NEL PAZIENTE ONCO-EMATOLOGICO NEUTROPENICO : TRATTAMENTO EMPIRICO VS. APPROCCIO PRESUNTIVO ("PRE-EMPTIVE").

Responsabile dello Studio : Dr. Giampaolo Bucaneve

Coordinatori : Dr. Giampaolo Bucaneve
Prof. Albano Del Favero
Dr. Francesco Menichetti
Dr.ssa Alessandra Micozzi

Open , multicenter, randomized trial comparing two therapeutic approaches for the treatment of invasive fungal infections in neutropenic onco-hematologic patients : empiric vs. presumptive (preemptive) treatment.

ABSTRACT

Background :Invasive fungal infections (IFI) are a major cause of morbidity and mortality in patients with neutropenia undergoing chemotherapy for cancer, as well as in other immunocompromised patients. In those patients the most commonly identified pathogens are Candida and Aspergillus species. Early treatment of invasive mould infections seems to improve the outcome but , because an early definitive diagnosis of IFI is difficult, empirical antifungal therapy (EAFT) has become a common practice in neutropenic patients with persistent or recurrent fever of unexplained origin (PFUO). EAFT was established as an adequate management of PFUO around 20 years ago with conventional amphotericin B deoxycholate (c-AmB). In recent years, EAFT trials for PFUO have used less toxic agents, such as the lipid formulations of AmB, the new azoles (Voriconazole, Posaconazole) and the echinocandins (caspofungin); these compounds provided similar efficacy with lower toxicity but at a much higher cost.
The empirical approach, which is often associated with an inadequate diagnostic work-up, unnecessarily treats patients who, despite fever, are not affected by invasive fungal infections, while, in the same time, may be inadequate for those patients who are actually affected by IFI.
Much recently, in some pilot trials, the use of new non-coltural based assaies tools (galactomannan and beta-D-glucan antigens) in conjunction with sensitive imaging techniques as CT scan and Bronchoalveolar lavage (BAL) showed to improve the rapid, early diagnosis of invasive fungal disease, drastically reducing the needs of empirical antifungals in favour of a "presumptive" (pre-emptive) therapeutic approach. Despite these promising observations the "presumptive" (pre-emptive) approach lacks validation in larger randomized clinical trial.
Objectives : To compare the empiric vs. the presumptive (pre-emptive) therapeutic approach in the treatment of IFI in neutropenic patients affected by acute leukemia and/or undergoing autologous Haematopoietic Stem Cell Transplantation (HSCT).
Methods: This is an open, multicenter, randomized trial . After an adequate (at least 4 days) broad spectrum antibiotic therapy, persistent febrile neutropenic patients, will be randomized to receive an empiric antifungal therapy or a delayed antifungal treatment started according to the results of a standardized work-up including at least the detection of the fungal antigens (galactomannan, beta-D-glucan, mannoprotein) an early and repeated pulmonary CT and BAL.
Expected results: Assuming from the literature a similar efficacy, the "presumptive" compared to the empirical approach would be able to reduce the use of antifungals with a possible reduction of the economic costs and toxicity, moreover the "presumptive" strategy using a standardized diagnostic work-up would be able to detect a more number of fungal infections than the empirical approach.

1. BACKGROUD AND RATIONALE

Invasive fungal infections (IFIs) are major causes of morbidity and mortality in neutropenic patients who receive chemotherapy for hematological malignancies or who undergo allogeneic hematopoietic stem cell transplantation[1–3]. Unfortunately, early diagnosis remains a challenge, given the low sensitivity of microbiological culture techniques and the low specificity of standard radiological tools, especially in neutropenic patients[4] . Therefore, infections are often far advanced at the time of diagnostic confirmation, and overall outcome is poor [5,6]. Empirical

antifungal therapy is considered standard practice in neutropenic patients with fever that persists or recurs while they are receiving broad-spectrum antibiotics and has repeatedly been endorsed by consensus guidelines [7, 8]. The aim is to ensure that patients with possible IFI receive therapy early in the course of the disease, because early initiation of therapy seems to improve the survival rate [9–15]

Empiric antifungal therapy was established as an adequate management of PFUO around 20 years ago with conventional amphotericin B deoxycholate (c-AmB) [9-10]. In recent years less toxic agents, such as the lipid formulations of AmB, the new azoles (Voriconazole, Posaconazole) and the echinocandins (caspofungin) has been used; these compounds compared to c-AmB provided similar efficacy with a lower toxicity but at a much higher cost [11-15].

Fever is a poorly predictive surrogate upon which to base an intervention such as empirical antifungal therapy [16]. Between 35% to 69% of leukaemia patients and 56% to 82% of HSCT receive empirical antifungal therapy, yet proven IFI occurs in only 2% to 15% [17-20] suggesting that the current guidelines [7] based upon persistent neutropenic fever are significantly flawed and may result in unjustifieable excess treatment-related toxicities and resource expenditures [16]. Therefore the empirical approach, which is often associated with an inadequate diagnostic work-up, unnecessarily treats patients who, despite fever, are not affected by invasive fungal infections, while, in the same time, may be inadequate for those patients who are actually affected by IFI.

Much recently, in some pilot trials, the use of new non-coltural based assay tools (galactomannan and beta-D-glucan antigens) in conjunction with sensitive imaging techniques as CT scan and Bronchoalveolar lavage (BAL) showed to improve the rapid, early diagnosis of invasive fungal disease, drastically reducing the needs of empirical antifungals in favour of a “presumptive” (pre-emptive) therapeutic approach. [21]. The objective of the “presumptive” strategy is to limit the unnecessary use of systemic antifungal drug therapy, reserving its use only for those patients who fail to respond to empiric antibiotics and present additional signs / symptoms indicative of IFI. In conclusion such a strategy targets the population in which there is sufficient evidence of pathogen invasion but no manifest symptomatic disease. The interest in the “presumptive” (pre-emptive) approach is also based upon the observation that early detection of IFI is associated with a better outcomes. A multicenter randomized study compared preemptive ant empirical antifungal therapy with liposomal amphotericin B among allogeneic HSCT recipients receiving fluconazole prophylaxis based on serial serum PCR fungal DNA detection studies [22]. The pre-emptive group received more antifungals than the mpirical therapy group (56% vs. 37% respectively; p <0,001). Although the numbers of documented IFI were similar (5,6% vs. 7.7%; p=0,0396) the 30 day mortality was reduced by 67% in the pre-emptive group (2,0% vs. 6,3%; p= 0,034).

A second study in acute leukaemia patients receiving fluconazole prophylaxis examined an algorithm-based pre-emptive approach based upon serial diagnostic testing and clinical monitoring [21]. Only patients with =/> 2 positive serum galactomannan assays or CT and or bronchoscopic evidence for mould infection received antifungal therapy. A total of 41 of 117 febrile neutropenic episodes (35%) had persistent neutropenic fever; however only 9 patients (22% of the 41 persistent neutropenic fevers; 8% of the original febrile neutropenic episodes) satisfied the pre-defined criteria for antifungal therapy , a 78% relative risk reduction in antifungal therapy use.

Despite these promising observations the “presumptive” (preemptive) approach lacks validation in larger randomized clinical trial.

This is an open, multicenter, randomized clinical trial comparing the effectiveness of a “presumptive” (preemptive) approach with the empirical antifungal strategy in the treatment of IFI in persistently febrile neutropenic patients.

Assuming from the literature for both therapeutic strategies a similar efficacy, the “presumptive” compared to the empirical approach would be able to reduce the use of antifungal therapy with a possible reduction of drug related toxicity and economic costs due to the use of antifungals in a more selected population. Moreover the “presumptive” strategy, using a standardized diagnostic work-up, would be able to detect a more number of fungal infections than the empirical approach.

2. OBJECTIVES OF THE STUDY

To compare the empiric vs. the presumptive (pre-emptive) therapeutic approach in the treatment of IFI in neutropenic patients affected by acute leukemia and/or undergoing autologous Haematopoietic Stem Cell Transplantation (HSCT).
Primary Objectives: To compare efficacy, safety and costs of "presumptive" treatment with empirical antifungal therapy in persistently febrile neutropenic acute leukemia and autologous HSCT patients.
Secondary Objectives :
To compare survival rates among patients treated with "presumptive" vs. empirical approach.
To compare clinical outcomes of patients treated with "presumptive" vs. empirical approach.
To evaluate the efficiency of a standardized WORK-UP in detecting IFIs.
To compare the use of other healthcare resources in patients treated with "presumptive" vs. empirical treatment.
To compare the duration of hospitalization (from the onset of the suspected IFI) in patients treated with "presumptive" vs. empirical treatment.

This trial tests the hypothesis that the "presumptive" strategy compared to the empirical approach has at least similar efficacy but it is related to a lower use of antifungals , a lower drug induced toxicity, a lower cost. Moreover, in the "presumptive" arm, the use of a standardized diagnostic work-up may be able to better detect invasive fungal infections in neutropenic oncohematologic patients.

3. METHODS

3.1 STUDY DESIGN

This is an experimental study. It is open, prospective, randomized, multicenter, conducted to compare the efficacy, safety, and tolerability of a "presumptive" (pre-emptive) antifungal treatment as opposed to empiric treatment of IFI in febrile neutropenic patients with hematological malignancies. The distinguishing characteristic of the "presumptive" approach is the use of a systematic diagnostic *WORK-UP* designed specifically to early reveal IFI and in this way to establish minimal requirements for initiation of antifungal therapy. See the study FLOW-CHART at ANNEX 1.

3.2 STUDY POPULATION

Febrile, chemotherapy induced neutropenic, Acute Leukemia (AL) or HSCT adult patients (age =/> 18 years) will be enrolled in this trial. All patients will be treated in Hospital.

3.2a.Inclusion Criteria

1. Sex: Male or female.
2. Age: ≥18 years.
3. Patients affected by acute leukemia (myeloid or lymphoid) or undergoing autologous HSCT.
4. Hospitalization for standard chemotherapy or autologous stem-cell transplantation.
5. Neutropenia : a neutrophil count of <500 cell/mm^3 or a count of <1000 cell/mm^3 that is expected to drop to <500 cell/mm^3 and remain at this level for at least 10 days [7].
6. Fever unresponsive to a previous adequate course of broad spectrum antibiotics (duration of therapy at least 96 hours):
    - *Fever:* a single measurement of body temperature ≥38.3°C or a temperature of ≥38.0°C lasting ≥1 hour [7].

7. Execution at least 96 hours before the randomization of an initial WORK-UP (Time 0) including at least : 3 blood specimens , 1 urine specimen and possibly 1 nasal swab, 1 pharyngeal swab, 1 rectal swab and excluding.
8. Written informed consent to all study procedures must be obtained from all patients.

3.2b Exclusion criteria

1. History of one or more documented or suspected IFIs prior to the current febrile episode.
2. Patients who have received allogeneic stem-cell transplantation.
3. History of hypersensitivity to or intolerance of antifungal drug administered after randomization
4. Concomitant treatment with other systemic antifungal drugs that can interfere with the evaluation of the study drugs.
5. Patients whose mental condition renders them incapable of understanding the nature, purpose, and possible consequences of the study AND/OR of adhering to the present protocol AND/OR those displaying uncooperative attitudes or behavior with respect to the study.
6. Patients with a life expectancy of less than 72 hours.
7. Patients with any condition that, in the investigator's judgment, could jeopardize the patient's safety during his or her participation in the study OR prevent evaluation of the patient's response to the study drug OR significantly reduce the likelihood that the patient will be able to complete treatment with the study drug assigned during the randomization process.
8. Patients with altered liver function indicated by SGPT (ALAT), SGOT (ASAT), total bilirubin or alkaline phosphatase ≥5 times the upper limit of normal ranges.
9. Patients with serum creatinine levels >1.5 x ULN (upper limit normal).
10. Pregnancy (Women).

3.2c Trial Design

If eligible, patients will be enrolled to receive either an early empirical antifungal therapy according to the IDSA 2002 guidelines [7] (empirical therapy arm) or a delayed antifungal therapy according to the results of a standardized WORK-UP (see ANNEX 2) aimed to ascertain an IFI ("presumptive" therapy arm).

Empirical treatment group :

In this arm after the initiation of antifungal therapy, patients will be assessed according to the IDSA 2002 guidelines [7]. Additional microbiological samples, diagnostic imaging of any organ suspected of having infection or other additional test will be assessed according to the Investigator's judgement.

"Presumptive" (pre-emptive) group :

In the "presumptive" therapy arm the antifungal treatment will be started if a diagnosis of probable, possible, definite invasive fungal infection will be made according to the EORTC-IFICG and NIAID-MSG criteria [23]. In an attempt to unnecessarily delaying effective treatment, this trial provides also for a diagnostic classification termed "FN +1," which will include all those febrile neutropenic patients ***(FN = patients with fever and neutropenia who fail to respond to empiric antibiotic therapy),*** whose diagnostic WORK-UP reveals at least one **(+ 1)** of the minimally accepted findings (clinical, microbiological, radiological or serological) for a suspicion of IFI listed in the ANNEX 3. The assignment to this category is the minimum requisite for the start of antifungal therapy in this group.

Therefore, in the "presumptive" arm the results of the standardized WORK-UP will allow the Investigator to make one of the following diagnoses, reflecting the likelihood of an IFI:

Class of diagnosis	Category	Definition
Class 1	FN	Patients with fever and neutropenia who fail to respond to empiric AB therapy and fail to meet the minimum requirements for evidence of IFI.
Class 2	FN + 1	**Appendix 2**
Class 3	Possible IFI	**Ascioglu et al CID 2002, [23]**
Class 4	Probable IFI	**Ascioglu et al CID 2002, [23]**
Class 5	Proven IFI	**Ascioglu et al CID 2002, [23]**

In any case, the total WORK-UP will last a minimum of 11 days.
At any time after randomization, if patient's clinical condition deteriorates the Investigator will be allowed to start antifungal therapy empirically.

If, at the end of the 11th day of the standardized WORK-UP, the patient still has a Class 1 (FN) diagnosis, the case may be managed in either of the following ways, at the discretion of the Investigator:
1 - The patient can continue the Work-up until it yields evidence sufficient for a Class 2 or higher diagnosis (in that case the antifungal treatment will be started), <u>OR</u> until the patient's neutrophil count rises to >500 cell/mm^3 (in this case the antifungal should not be started).
2 - Alternatively, if patient's conditions are deteriorating, the diagnostic WORK-UP can be terminated, and the FN patient can be treated empirically with any antifungal regimen deemed appropriate by the Investigator.

4. INTERVENTIONS

4.1 Empiric Antibiotic Therapy

At onset of fever (Time 0) each neutropenic oncohematologic patient will be assessed for clinical signs and symptoms of infection and the following specimens should be cultured :
3 blood cultures (one from the central venous line if applicable)
1 urine culture
1 nasal swab
1 pharingeal swab
1 rectal swab
Empiric antibiotic therapy should be started according to IDSA 2002 guidelines [7] and it should be continued for at least 96h. After this minimum required time, if fever persist and an infection is not clinically or microbiologically documented the patient could be randomized either to receive, as soon as possible, an empiric antifungal therapy or to start a standardized WORK-UP (see Apendix) aimed to document an IFI before the start of the antifungal treatment.
The diagnostic studies included in the standard WORK-UP are listed in the APPENDIX 1.

4.2 Antifungal Therapy

- In the empiric treatment arm, the antifungal therapy will be started as soon as possible if fever will persist despite an adequate course (at least 96 hours) of a broad spectrum antibiotic regimen.
- In the "presumptive" treatment arm, the antifungal therapy will be started at any time a FN+1, probable, possible or definite invasive fungal infection is documented according to the IATCG . NIAID-MSG criteria [23], and the criteria stated in the APPENDIX 2.

The initial antifungal regimen should be the same in the two treatment arms; eligible drugs should be:
Ambisome: 3mg kg/die, IV

Abelcet : 5 mg/Kg/die, IV
Caspofungin : 50 mg/die IV

- In the empiric therapy arm, the minimum duration of antifungal therapy will be 2 weeks. The treatment should be continued at least for 4 weeks in case of a probable or definite IFI. In this case the first line antifungal regimen would be modified to target the causative fungal agent.
- In the "presumptive" therapy arm the minimum duration of treatment will depend on the Diagnostic Class the patient has been assigned based on the results of the standardized WORK-UP. For patients classified as Class 2 (FN+1) or 3 (Possible infection) antifungal therapy will be continued for at least 2 weeks, for those classified as Class 4 (Probable infection) or 5 (Definite) antifungal therapy will be continued al least for 4 weeks.

4.3 Patient Assessment

All randomized patient will be clinically assessed daily. They will be evaluated for a total of 4 weeks after the End of Treatment (ET).

After randomization all patients will undergo a series of examinations and tests.

4.3a All Randomized Patients

For all randomized patients the following evaluations and tests will be carried out at the times specified below:

Daily

- Clinical signs and symptoms:
  fever
  headache
  hemoptysis
  cough
  sputum production
  chest pain
  dyspnea
  facial/sinus pain
  skin lesions
  other

Each sign or symptom will be rated as grade 0 (absent), 1 (mild), 2 (moderate), or 3 (severe), according to the National Cancer Institute classification (See **Appendix 9**).

Weekly

- Blood and urine for laboratory safety tests.
- Adverse experience: elicit and record any adverse events since previous visit.
- Length of hospitalization, level of patient care and details of outpatient care.
- In case of withdrawal of study treatment: it must be decided by the principal investigator.

4.3b Patients enrolled in the Empiric therapy arm

They will be assessed following the IDSA 2002 Guidelines [7], according to the judgment of the investigator. All the results of additional examinations and the patient's clinical outcome will be recorded.

In case of IFI (possible, probable, definite) the timing and type of subsequent examinations will be decided by the investigator.

4.3c Patients enrolled in the "Presumptive" therapy arm

They will be assessed according to a standardized WORK UP listed in APPENDIX 1.

If the minimum criteria to start antifungal therapy will be reached, the additional following investigations should be carried out according to the Diagnostic Class:

Patients in the Diagnostic Class 2 (FN+1) or 3 (Possible IFI)

Every other day for the duration of treatment:

- Serology for galactomannan (for *Aspergillus* spp.)
- Serology for mannoprotein (for *Candida* spp.)

Once a week for the duration of treatment:

- CT-scan of the region involved in the infection.
- Cultures of previously infected sites.
- Other instrumental or microbiological studies considered necessary by the Investigator.

Patients in the Diagnostic Class 4 (Probable IFI) or 5 (definite IFI)

Two times / week for the duration of treatment

- Serology for galactomannan (in patients infected with *Aspergillus* spp.).
- Serology for mannoprotein (in patients infected with *Candida* spp.) (for descriptive purposes only).

Weeks 4, EOT, 4 weeks after the EOT

- Repeat cultures of all previously infected sites (if possible).
- CT and/or ultrasonography of the region involved in the infection.

However in all randomized patients reaching a diagnosis of probable or proven invasive fungal infection the patient’s care may require an higher-frequency assessment than indicated in the study protocol and in case this will be guaranteed by the investigator.

5. OUTCOMES

5.1 EFFICACY PARAMETERS

The following efficacy parameters will be evaluated between the two treatment groups :

- Number of patients undergoing antifungal therapy
- Number of documented IFI
- Response to antifungal therapy
- Outcome
- Safety
- Direct costs

5.2 Response to Antifungal Therapy

5.2a All Patients

For all patients, the Primary Overall Response will be evaluated at the End Of Treatment (EOT), OR when the patient has completed 12 weeks of treatment.

Treatment will be considered successful if the patient will be compliant with ALL of the following criteria:

- No breakthrough fungal infections within 7 days of end of therapy

- Survival 7 days after end of therapy
- No discontinuation due to toxicity or lack of efficacy
- Resolution of fever
- Complete or partial response of patients with base-line fungal infections by 12 weeks/end of treatment.

In patients classified in the Diagnostic Class 2 (FN+1), defervescence without any other clinical sign attributable to IFI will be the parameters considered for a favourable clinical response.

5.2b Patients with a diagnosis of Probable or Definite IFI

Moulds infections

All patients with a diagnosis of Probable or Definite Invasive Fungal Infection sustained by moulds will be additionally evaluated for efficacy according to the following criteria adopted by Herbrecht R. et al. for documented mould fungal infections [24].

- Clinical Response *(to be evaluated at Weeks 1, 2, 4, EOT/12 weeks and 4 weeks after the EOT)*
- Radiological Response *(to be evaluated at Weeks 4, EOT/12weeks and 4 weeks after the EOT)*
- Microbiological Response *(to be evaluated at Weeks 4, EOT/12 weeks and 4 weeks after the EOT)*
- Overall Response and Global Response *(to be evaluated at the EOT/12 weeks and 4 weeks after the EOT)*

Yeasts infections

All patients with a diagnosis of Probable or Definite Fungal Infection sustained by yeast will be additionally evaluated for efficacy according to criteria adopted by Mora-Duarte J. et al. [24].

- Favorable overall response
- Unfavorable overall response

5.3 Outcome

The deaths occurring during the study period will be reported for each treatment group. If available, the results of the autopsy should be recorded.

The causative relationship between the death and a fungal infection should be assessed and will be classified into one of the following 4 categories:

1. Death caused by IFI.
2. Death unrelated to IFI, but evidence of active fungal infection present.
3. Death unrelated to IFI, and no evidence of residual fungal infection.
4. Indeterminate

The following survival analyses will be also performed:
Time to start of antifungal therapy
Time to discontinuation of antifungal therapy
Time to death

5..4 Safety

The occurrence of Adverse Reactions (AR) related to antifungal treatments will be monitored and recorded for each treatment group as well as the withdrawal due to ARs. Safety data will be submitted to clinical review. The rates of ARs and other safety related variables will be tabulated according to treatment group and reported.

5.5 Direct Costs

An exploratory assessment of resource utilization will be conducted in both arms over the study period. Collected data will be tabulated according to treatment group and will be summarized by appropriate descriptive statistics.

5. SAMPLE SIZE ESTIMATES

Although definitive data are not available in this field, we assume that about 30% of febrile neutropenic patients fail to respond to a first line broad spectrum empiric antibiotic therapy and therefore are eligible to start empiric antifungal therapy according to the IDSA 2002 Guidelines. A sample size of 242 patients (121 per group) will be needed to demonstrate between the two study groups a 20% difference in the use of antifungal therapy, assuming a beta-error=0.20 ed an alfa-error=0.05. On the assumption of a 10% rate of not available patients a total number of 266 patients (133 for each treatment arm) should be enrolled.

6. RANDOMIZATION

Patients will be randomized centrally only once, according to a computer-generated random-number program accessible 24 hours daily. Patients will be stratified according to the center.

7. INFORMATION RETRIVAL

The patient's personal and clinical data together with the information on the use of and response to antifungal therapy and outcome will be recorded in a case report form which will be filled by the investigator of each participating center. The data will be subsequently recorded on a computerized data-base for analysis.

8. STATISTICAL ANALYSIS

All case report form will be centrally reviewed and data analysis (SAS, SAS Insitute) will be blinded to assigned treatment. A by-protocol analysis on the assessable patients and an intent-to-treat analysis on all eligible patients will be performed. The χ-square test with a correction for continuity and Fisher's exact test will be used , when appropriate. The Wilcoxon test will be used to compare the means. The distributions time-to-event variables will be calculated by the Kaplan-Meier method and will be compared by the log-rank test. The 95% Confidence Intervals for the difference between proportions will be given when appropriate.

A logistic regression model will be used to assess the relative importance of the various prognostic factors assessable at the time of randomization. The OR of success and its 95% CI will be calculated for each factor included in the multivariate analysis model.

An exploratory assessment of resource utilization will be conducted in both arms over the study period. Collected data will be tabulated according to treatment group and will be summarized by appropriate descriptive statistics.

9. ORGANIZATIONAL CHARACTERISTICS AND FEASIBILITY

This is a multicenter study. Hematologic Units will represent the majority of participating Centers. All the participating centers are members of the GIMEMA (Gruppo Italiano Malattie Ematologiche dell'Adulto) Infection Program; the majority of the investigators have already participated at least one of the published GIMEMA infection trials ( ). The list of those Centers which have already agreed to this study is summarized in the ANNEX 4.

The coordinating Center will be the Istituto di Medicina Interna e Scienze Oncologiche dell'Azienda Ospedaliera di Perugia which will be involved in the monitoring activities, the computer centralized randomization system, the data managing and analysis. The same Units provided to coordinate all the trials published by the GIMEMA Infection program in the last two decades.

A monitoring Committee and a Data Review Committee will be set up by the GIMEMA (Gruppo Italiano Malattie Ematologiche dell'Adulto) Infection Program.. The committee will review, in a blind fashion the collected data according to the end-points/outcomes defined by the protocol.

10. TIMING

The duration of the study will be 2 years. A first check point for the evaluation of work in progress will be planned at the end of the first year and at the end of the enrollment phase.

Data will be primary reviewed by the Data Review Committee . A final report will be provided at the end of data analysis.

11. GOOD CLINICAL PRACTICES AND ETHICAL ASPECTS

This study will be conducted in accordance with Good Clinical Practices.

The safety of the “presumptive” approach has been verified only in not randomized, open trials. In those studies the presumptive methodology showed to be as effective as the empiric antifungal approach. However pre-definite clinical reassessment check-points to monitor possible patient clinical deterioration and the standardized WORKUP are planned for this study to allow a prompt initiation of antifungal therapy also in the “presumptive” treatment arm.

12. REFERENCES

1. Marr KA, Carter RA, Boeckh M, et al. Invasive aspergillosis in allogeneic stem cell transplant recipients: changes in epidemiology and risk factors. Blood 2002; 100:4358–66.
2. Denning DW. Invasive aspergillosis. Clin Infect Dis 1998; 26:781–803.
3. Herbrecht R, Neuville S, Letscher-Bru V, et al. Fungal infections in patients with neutropenia: challenges in prophylaxis and treatment. Drugs Aging 2000; 17:339–51.
4.Hope WW, Denning DW. Invasive aspergillosis: current and future challenges in diagnosis and therapy. Clin Microbiol Infect 2004; 10:2–4.
5.Patterson TF, Kirkpatrick WR, White M, et al. Invasive aspergillosis: disease spectrum, treatment practices, and outcomes. Medicine 2000; 79: 250–60.
6. Herbrecht R, Denning DW, Patterson TF, et al. Voriconazole versus amphotericin B for primary therapy of invasive aspergillosis. N Engl J Med 2002; 347:408–15.
7.Hughes WT, Armstrong D, Bodey GP, et al. 2002 Guidelines for the use of antimicrobial agents in neutropenic patients with cancer. Clin Infect Dis 2002; 34:730–51.
8. Link H, Bohme A, Cornely OA, et al. Antimicrobial therapy of unexplained fever in neutropenic patients—guidelines of the Infectious Diseases Working Party (AGIHO) of the German Society of Hematology and Oncology (DGHO), study group interventional therapy of unexplained fever, Arbeitsgemeinschaft Supportivmassnahmen in der Onkologie (ASO) of the Deutsche Krebsgesellschaft (DKG- German Cancer Society). Ann Hematol 2003; 82(Suppl 2):S105–17.
9. Pizzo PA, Robichaud KJ, Gill FA, Witebsky FG. Empiric antibiotic and antifungal therapy for cancer patients with prolonged fever and granulocytopenia. Am J Med 1982; 72:101–11.
10. EORTC International Antimicrobial Therapy Cooperative Group. Empiric antifungal therapy in febrile granulocytopenic patients. Am J Med 1989; 86:668–72.
11. Prentice HG, Hann IM, Herbrecht R, et al. A randomized comparison of liposomal versus conventional amphotericin B for the treatment of pyrexia of unknown origin in neutropenic patients. Br J Haematol 1997; 98:711–8.
12. Walsh TJ, Finberg RW, Arndt C, et al. Liposomal amphotericin B for empirical therapy in patients with persistent fever and neutropenia. National Institute of Allergy and Infectious Diseases Mycoses Study Group. N Engl J Med 1999; 340:764–71.
13. Boogaerts M,Winston DJ, Bow EJ, et al. Intravenous and oral itraconazole versus intravenous amphotericin B deoxycholate as empirical antifungal therapy for persistent fever in neutropenic patients with cancer who are receiving broad-spectrum antibacterial therapy: a randomized, controlled trial. Ann Intern Med 2001; 135:412–22.
14. Walsh TJ, Pappas P, Winston DJ, et al. Voriconazole compared with liposomal amphotericin B for empirical antifungal therapy in patients with neutropenia and persistent fever. N Engl J Med 2002; 346:225–34.
15. Walsh TJ, Teppler H, Donowitz GR, et al. Caspofungin versus liposomal amphotericin B for empirical antifungal therapy in patients with persistent fever and neutropenia. N Engl J Med 2004; 351:1391–402.
16. De Pauw BE. Between over- and undertreatment of invasive fungal disease. Clin Infect Dis. 2005;41:1251-1253.
17. Bow EJ, Laverdiere M, Lussier N, et al. Antifungal prophylaxis for severely neutropenic chemotherapy recipients: a meta analysis of randomized-controlled clinical trials. Cancer. 2002;94:3230-3246.
18. Cornely O, Maertens J, Winston D, et al. Posaconazole vs standard azole (FLU/ITRA) therapy for prophylaxis of invasive fungal infections (IFIs) among high-risk neutropenic patients: results of a randomized, multicenter trial. ASH Annual Meeting Abstracts. 2005;106:1844.

19. Ullmann AJ, Lipton JH, Vesole DH, et al. A multicenter trial of oral posaconazole vs. fluconazole for the prophylaxis of invasive fungal infections in recipients of allogeneic hematopoietic stem cell transplantation with graft-vs.-host disease [abstract]. Interscience Conference on Antimicrobial Agents and Chemotherapy 2005.
20. Kanda Y, Yamamoto R, Chizuka A, et al. Prophylactic action of oral fluconazole against infection in neutropenic patients—a meta-analysis of 16 randomized, controlled trials. Cancer. 2000;89:1611-1625.
21. Maertens J, Theunissen K, Verhoef G, et al. Galactomannan and computed tomography-based preemptive antifungal therapy in neutropenic patients at high risk for invasive fungal infection: a prospective feasibility study. Clin Infect Dis. 2005;41:1242-1250.
22. Hebart H, Klingspor L, Klingebiehl T, et al. PCR-based liposomal amphotericin B treatment following allogeneic stem cell transplantation is a safe treatment strategy: preliminary results of a prospective study. ASH Annual Meeting Abstracts. 2004;104:192.
23. Ascioglu S. et al. Defining opportunistic invasive fungal infections in immunocompromised patients with cancer and hematopoietic stem cell transplants: an international consensus. Clin Infect Dis. 2002 ; 1;34(1):7-14.
24. Herbrecht R. et al. Voriconazole versus amphotericin B for primary therapy of invasive aspergillosis. N Engl J Med. 2002 ; 8;347(6):408-15.
25. Mora-Duarte J. et al. Comparison of caspofungin and amphotericin B for invasive candidiasis. NEJM 2002; 19;347(25):2020-9.

ANNEX 1

Adult (=/>18 years) Acute Leukemia and/or Hematopoietic Stem Cell Transplanted patients with Fever unresponsive to an adequate (at least 96 hours) empiric broad spectrum antibiotic therapy. :

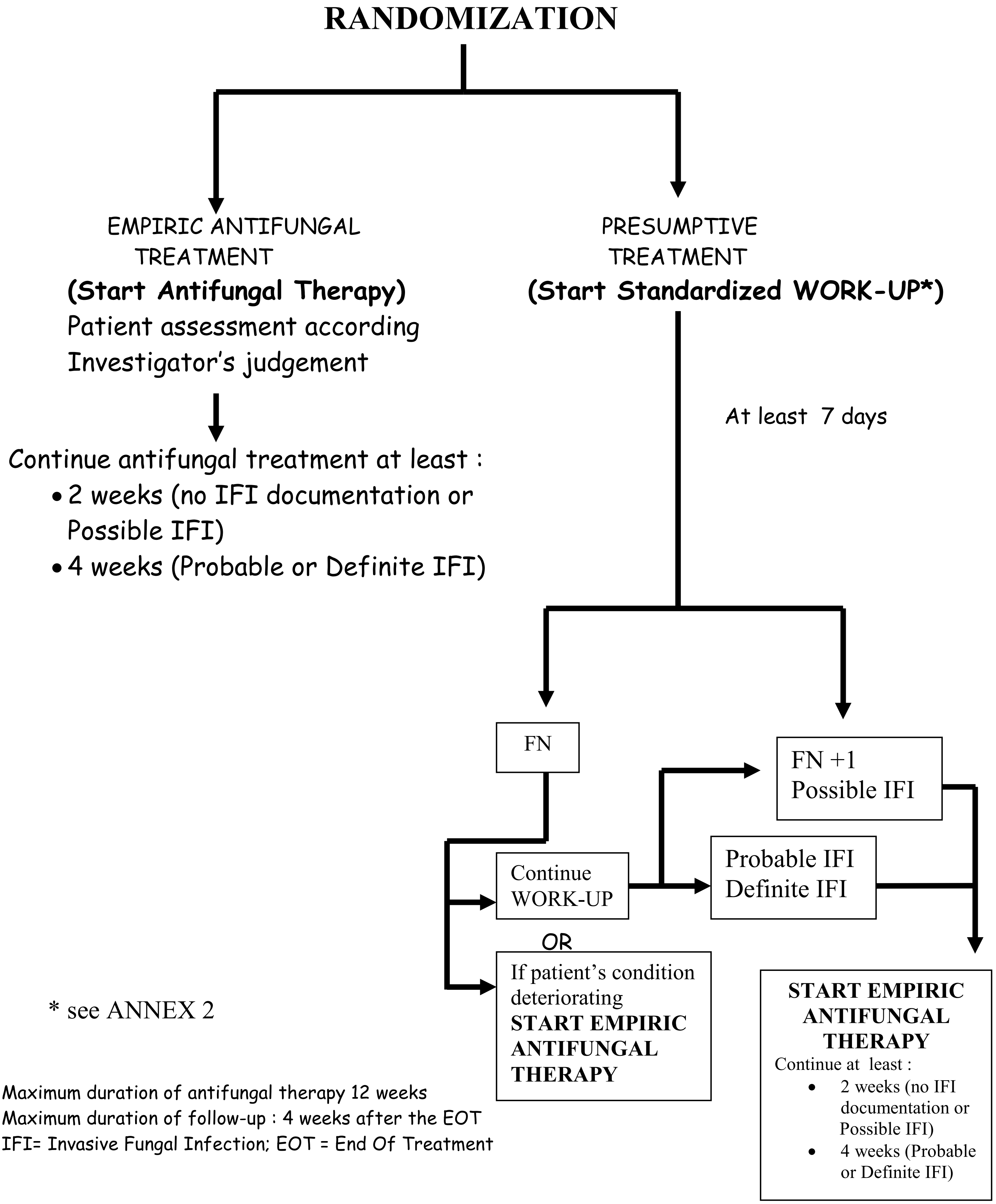

* see ANNEX 2

Maximum duration of antifungal therapy 12 weeks
Maximum duration of follow-up : 4 weeks after the EOT
IFI= Invasive Fungal Infection; EOT = End Of Treatment

ANNEX 2

Definition of Standardized WORK-UP

Examination	Timing and procedures
**Thoracic CT scan**	The study will be considered positive when it reveals one of the major or minor signs of IFI involving the lower respiratory tract, as defined by the EORTC-IFICG and NIAID-MSG (Ascioglu S. et al., CID 2002).<br>If the first scan is negative, scans can be repeated at 3-day intervals.
**3 blood cultures**	Fungal cultures; repeat as indicated
**Galactomannan serology (for *Aspergillus*)**	Blood specimens collected daily.
**Mannoprotein serology (for *Candida*)**	Blood specimens collected daily.
**Nasal swab culture (for *Aspergillus*)**	Repeat as indicated.
**Pharyngeal swab culture (for *Candida*)**	Repeat as indicated
**Rectal swab culture (for *Candida*)**	Repeat as indicated
**Urine culture (for *Candida* and other yeasts)**	Repeat as indicated

ANNEX 3

Definition of patients – FN + 1 –

This category includes febrile neutropenic (FN) patients who fail to respond to empiric antibiotic therapy AND present at least one of the following findings:

A. One nasal swab culture that is positive for moulds (with the exclusion of Zygomycetes, *A. terreus* and *Scedosporium* spp.).

B. Culture positivity indicative of colonization by *Candida* species and/or other yeast species at two non-adjacent body sites, e.g., pharyngeal swab + urine or pharyngeal swab + rectal swab or nasal swab + urine. The concomitant colonization of two adjacent body sites (e.g., urine specimen + rectal swab positivity or nasal + pharyngeal swab positivity) is not sufficient to satisfy this criterion.

C. Positivity for the *Candida* antigen, mannoprotein, in a single blood specimen.

D. Positivity (≥ 0.7) for the *Aspergillus* antigen, galactomannan, in a single blood specimen.

E. Evidence of *Candida* enterocolitis or ileotyphlitis manifested by the simultaneous presence of abdominal pain AND diarrhea (more than three bowel movements / day) AND ultrasound findings of colon-wall thickening (wall thickness: >5 mm).

F. A single thoracic CT scan showing findings compatible with a new pulmonary infiltrate other than those listed as "major criteria" by the EORTC-IFICG and NIAID-MSG (Ascioglu S. et al., CID 2002[23]).

G. A single CT scan of the nasal sinuses showing findings compatible with early-stage sinusitis other than those listed as "major criteria" by the EORTC-IFICG and NIAID-MSG (Ascioglu S. et al., CID 2002 [23]).

H. A single cerebral CT scan showing findings compatible with an intraparenchymal mass lesion other than those listed as "major criteria" by the EORTC-IFICG and NIAID-MSG (Ascioglu S. et al., CID 2002[23]).

ANNEX 4

GIMEMA CENTRES WHICH ALREADY AGREED WITH THE STUDY

1. DIVISIONE EMATOLOGIA, AZ. OSPEDALIERA SS ANTONIO E BIAGIO, ALESSANDRIA
2. S.C. Ematologia e Trapianto Ematopoietico, AZ. OSPEDALIERA S.G. MOSCATI, AVELLINO
3. CENTRO DI RIFERIMENTO ONCOLOGICO, Istituto Nazionale Tumori AVIANO, (PN)
4. EMATOLOGIA, UNIVERSITA' -POLICLINICO-AZ. MISTA OSP/UNIV, BARI
5. EMATOLOGIA, OSPEDALI RIUNITI DI BERGAMO, BERGAMO
6. IST. Ematologia e Oncologia Medica, POLICLINICO S.ORSOLA, BOLOGNA
7. U.O. EMATOLOGIA, AZIENDA OSPEDALIERA "PUGLIESE-CIACCIO", CATANZARO
8. U.O. EMATOLOGIA, AZ. OSP. UNIV. CAREGGI, FIRENZE
9. SC Ematologia Ospedali Riuniti Foggia - AOU FOGGIA
10. Reparto di Onco-Ematologia, Ospedale Filettino, LA SPEZIA
11. U.O.C. EMATOLOGIA, OSPEDALE S.MARIA GORETTI, LATINA
12. S.C. di EMATOLOGIA, OSPEDALE NIGUARDA Cà GRANDA, MILANO
13. EMATOLOGIA I, CTMO, OSPEDALE MAGGIORE IRCCS, MILANO
14. DIV. EMATO-ONCOLOGICA, ISTITUTO EUROPEO DI ONCOLOGIA, MILANO
15. Ematologia Policlinico di Modena, MODENA
16. DIV. EMATOLOGIA, AZIENDA OSPEDALIERA di RILIEVO NAZIONALE, NAPOLI
17. DIV. EMATOLOGIA OSP. S GIOVANNI BOSCO ASL NA 1 NAPOLI
18. DIV. DI EMATOLOGIA, AZ. Osp. UNIVERSITARIA, POLICLINICO FEDERICO II , NAPOLI
19. UOSC Ematologia-Oncologia, Istituto nazionale Tumori "Pascale", NAPOLI
20. Divisione di Medicina Interna II ed Ematologia ASO S.Luigi Gonzaga, ORBASSANO, (TO)
21. DIVISIONE DI EMATOLOGIA con TRAPIANTO AZIENDA OSPEDALIERA "V. CERVELLO" PALERMO
22. DIV. DI EMATOLOGIA E TMO A.U. POLICLINICO, PALERMO
23. EMATOLOGIA e CTMO OSPEDALE DI PARMA PARMA
24. IST.MED.INT.E SCI.ONCOL. ED EMATOLOGIA AZIENDA OSPEDALIERA DI PERUGIA, PERUGIA
25. U.O. di Ematologia Ospedale Civile di Pescara PESCARA
26. DIPART. ONCO-EMATOLOGICO AZ. USL RAVENNA, OSPEDALE S.MARIA DELLE CROCI ,RAVENNA
27. DIP.TO Ematologia AZ. OSP. "Bianchi-Melacrino-Morelli", REGGIO CALABRIA
28. UOC Ematologia, Ospedael S. Eugenio Università Tor Vergata ROMA
29. Az. Osp. Sant'Andrea II FACOLTA' DI MEDICINA, UNIVERSITA' "LA SAPIENZA", ROMA
30. Ematologia e Trapianti di Cellule Staminali Azienda Ospedaliera S. Camillo Forlanini ,ROMA
31. DIV. EMATOLOGIA UNIV. CATTOLICA del SACRO CUORE, ROMA
32. Ematologia Osp. S. Eugenio Università Tor Vergata, ROMA
33. IST. EMATOLOGIA, DIP. DI BIOPATOLOGIA UNIVERSITA' "LA SAPIENZA", ROMA
34. Sez. di Ematologia Ist. Clinico Humanitas ,ROZZANO (MI)
35. DIV. DI EMATOLOGIA, Osp. "Casa Sollievo della Speranza" IRCCS , S. GIOVANNI ROTONDO(FG)
36. ISTITUTO DI EMATOLOGIA Università di Sassari SASSARI
37. CLINICA DI EMATOLOGIA OSPEDALE RIUNITI TORRETTE DI ANCONA (AN)
38. SC Ematologia Presidio Ospedaliero di Treviso TREVISO
39. CLINICA EMATOLOGICA Policlinico Universitario UDINE
40. U.O. di Ematologia Ospedale Umberto I° MESTRE – VENEZIA
41. DIV. DI EMATOLOGIA ULSS N. 6 Ospedale San Bortolo VICENZA“
