# Supplementary material for: Pre-emptive versus empirical antifungal therapy in patients with febrile neutropenia with acute leukaemia: a GIMEMA study
Source: JAC Antimicrob Resist. 2026 Jun 26;8(3):dlag120. doi: 10.1093/jacamr/dlag120 (PMC13308701; doi:10.1093/jacamr/dlag120)
Supplement: dlag120_Supplementary_Data [file dlag120_supplementary_data.zip › Supplementary data JACAMR2025 543.R1.docx]

**Supplementary data**

**Figure S1.** Patient enrolment and disposition (CONSORT diagram)

**Patients Randomly assigned (n=101)**

**EMPIRIC (n=42)**

**Received allocated intervention (n=42)**

**PRE-EMPTIVE (n=59)**

**Received allocated intervention (n=59)**

**Analyzed (n=52)**

**Excluded from Analysis (n=7**)**

** *Not Eligible: 1; Protocol Violation: 6*

**lost to follow-up (n=0)**

**discontinued intervention (n=0)**

**Analyzed (n=40)**

**Excluded from Analysis (n=2*)**

** Not Eligible: 2*

**Lost to follow-up (n=0)**

**Discontinued intervention (n=0)**

**Lost to follow-up (n=0)**

**Discontinued intervention (n=0)**

**Table S1.** Patient characteristics (per protocol population)

| **Characteristics** | **Empiric**  **n=40** | **Preemptive**  **n=52** | **CI (95%)**  **from difference** | **p-value** |
| --- | --- | --- | --- | --- |
| **Male/Female** | 24/16 | 20/32 | 0.21 (0.01 to 0.41) | 0.06 |
| **Mean age (range)** | 56 (21-84) | 56 (19-80) | 0 (-5.8 to 5.8) | 0.8 |
| **Underlying disease**  *Acute myeloid leukemia*  *Acute lymphoblastic leukemia*  *Other acute leukemia* | 37/40(92%)  2/40 (5%)  1/40 (2%)^*^ | 46/52 (88%)  5/52 (10%)  1/52 (2%)^#^ | 0.04 (-0.07 to 0.16)  -0.05 (-0.15 to 0.05)  0.005 (-0.05 to 0.06) | 0.3  0.3  0.6 |
| **Chemotherapy**  *Remission induction/reinduction*  *Relapse/Reinduction*  *Remission Consolidation*  *Autologous Stem Cell Transplant* | 24/39** (61%)  9/39 (23%)  2/39 (5%)  4/39 (10%) | 36/52 (69%)  9/52 (17%)  4/52 (8%)  3/52 (6%) | -0.02 (-0.20 to 0.17)  0.09 (-0.08 to 0.25)  -0.01 (-0.07 to 0.09)  0.04 (-0.06 to 0.15) | 0.9  0.4  0.5  0.3 |
| **Protective environment**  *Multiple bedded room*  *Single room/reverse isolation*  *Laminar Air Flow Room* | 15/39** (38%)  4/39 (10%)  20/39 (52%) | 18/52 (35%)  4/52 (8%)  29/52 (57%) | 0.03 (-0.16 to 0.23)  0.02 (-0.09 to 0.14)  -0.05 (-0.25 to 0.16) | 0.8  0.4  0.8 |
| **Antibacterial prophylaxis**  *No*  *Ciprofloxacin*  *Levofloxacin*  *Other* | 4/40 (10%)  23/40 (57%)  11/40 (27%)  2/40 (5%) | 7/52 (13%)  22/52 (43%)  20/52 (38%)  3/52 (6%) | -0.03 (-0.16 to 0.08)  0.14 (-0.05 to 0.35)  -0.11 (-0.30 to 0.08)  0.01 (-0.1 to 0.08) | 0.4  0.2  0.3  0.6 |
| **Antifungal prophylaxis**  *Posaconazole*  *Other azoles (Fluconazole or Itraconazole)*  *no prophylaxis* | 23/40 (58%)  12/40 (30%)  5/40 (12%) | 28/52 (54%)  18/52 (35%)  6/52 (11%) | 0.06 (-0.14 to 0.26)  -0.004 (-0.23 to -0.14)  -0.009 (-0.12 to 0.14) | 0.7  0.8  0.5 |
| **Antiviral prophylaxis**  *Acyclovir*  *Other*  *no prophylaxis* | 13/39** (33%)  1/39 (3%)*^§^*  25/39 (64%) | 19/48§§ (40%)  1/48 (2%)^  28/48 (58%) | 0.07 (-0.26 to 0.14)  0.004 (-0.05 to 0.06)  -0.04 (-0.12 to 0.03) | 0.8  0.6  0.7 |
| **Mean duration of neutropenia –** days (range)  < 1,000/mmc  <100/mmc - days | 24 (3-53)  9 (1-17) | 27 (4-69)  10 (1-28) | -3 (-8.05 to 2.05)  -1 (-3.9 to 2.5) | 0.2  0.6 |
| **Neutropenia at randomization**  *<100/mmc* | 32/39** (82%) | 40/50 (80%) | 0.02 (-0.14 to 0.18) | 0.9 |
| **Total duration of fever - days (range)** | 8 (1-17) | 9 (1-28) | -1 (-2.8 to 0.97) | 0.3 |
| **Length of hospitalization from suspected IFI -** days (range) | 35 (15-98) | 38 (12-87) | 3 (-10.3 to 3.3) | 0.3 |

^*^Leukemic lymphoma

^#^Plasma cell leukemia

*^§^*Lamivudine

^Foscarnet

IFI: invasive fungal infection

**in one patient, chemotherapy, protective environment, and antiviral prophylaxis data were not available, the percentage was calculated based on 39 patients.

§§ In 4 patients, antiviral prophylaxis data were not available, and the percentage was calculated based on 48 patients.

**Table S2.** Forward stepwise regression on independent factors possibly associated with the initiation of antifungal therapy

|  | **Start of Antifungal Therapy** | **Univariate**  **(p)** | **Logistic Regression (p)** |
| --- | --- | --- | --- |
| **Age (years)** |  |  |  |
| <60 | 38/50 (76%) | 0.8 | 0.9 |
| ≥60 | 32/42 (76%) |  |  |
| **Sex** |  |  |  |
| M | 38/44 (86%) | 0.04 | 0.09 |
| F | 32/48 (69%) |  |  |
| **Duration of neutropenia at randomization (days)** |  |  |  |
| ≥ 10 days | 32/38 (84%) | 0.2 | 0.07 |
| < 10 days | 38/53 (72%) |  |  |
| **Type of Antifungal Prophylaxis** |  |  |  |
| Posaconazole | 38/51 (74%) | 0.8 | 0.5 |
| Others | 33/41 (80%) |  |  |
| **Antifungal Therapeutic Strategy** |  |  |  |
| Empiric | 40/40 (100%) | 0.000 | <0.01 |
| Preemptive | 30/52 (58%) |  |  |

**Table S3** Proven and probable IFIs: patients’ characteristics

| **Patient’s**  **initials** | **Assigned strategy** | **Oral Posaconazole prophylaxis** | **IFI Classification** | **Site of infection** | **Diagnostics** | **No. of positive galactomannan tests ^*^** | **Highest galactomannan result** | **Patterns on CT scan** | **Agent**  **(highly suspected)** | **Antifungal therapy** | **Outcome** |
| --- | --- | --- | --- | --- | --- | --- | --- | --- | --- | --- | --- |
| DI MA | Empiric | yes | Probable IFI | Lung | CT scan and positive galactomannan test | 2 | 0.7 | Two pulmonary lesions with air crescent sign | (*Aspergillus spp*) | Amphotericin B lipid complex, continued with Voriconazole | Survived |
| FA RI | Preemptive | yes | Proven IFI | Lung and blood | CT scan, sputum and blood cultures | - | - | Multiple bilateral pulmonary nodular lesions | *Geotrichum capitatum* | Liposomal Amphotericin B | Survived |
| FA GR | Preemptive | yes | Probable IFI | Lung | CT scan and positive galactomannan test | 2 | 0.5 | Pulmonary well-circumscribed lesion | (*Aspergillus spp*) | Liposomal Amphotericin B continued with Voriconazole | Survived |
| BU GI | Preemptive | yes | Probable IFI | Lung | CT scan and positive galactomannan test | 2 | 0.5 | Multiple bilateral well-circumscribed pulmonary lesions | (*Aspergillus spp*) | Liposomal Amphotericin B continued with Voriconazole | Survived |
| MO LU | Preemptive | no | Proven IFI | Blood | Blood cultures | - | - | - | *Candida glabrata* | Amphotericin B lipid complex continued with Liposomal Amphotericin B | Survived |
| DA RO | Preemptive | no | Probable IFI | Lung | CT scan and positive galactomannan test | 1 | 0.7 | Multiple bilateral well-circumscribed pulmonary lesions with halo sign | (*Aspergillus spp*) | Voriconazole | Survived |
| TR MA | Preemptive | no | Probable IFI | Lung | CT scan and positive galactomannan test | 3 | 0.5 | Multiple bilateral pulmonary nodular lesions | (*Aspergillus spp*) | Liposomal Amphotericin B | Survived |
| DE TO | Preemptive | no | Probable IFI | Lung | CT scan and positive galactomannan test | 5 | 1.7 | Multiple bilateral pulmonary lesions with air crescent sign | (*Aspergillus spp*) | Liposomal Amphotericin B continued with Voriconazole | Survived |
| RI ST | Preemptive | no | Probable IFI | Lung | CT scan and positive galactomannan test | 1 | 0.7 | Multiple bilateral pulmonary lesions with air crescent sign | (*Aspergillus spp*) | Liposomal Amphotericin B continued with Voriconazole | Survived |
| LI IN | Preemptive | no | Probable IFI | Lung | CT scan and positive galactomannan test | 1 | 0.7 | Multiple bilateral pulmonary lesions with halo sign | (*Aspergillus spp*) | Voriconazole | Survived |

**Table S4** Preemptive group: cases classified as FN+1 at standardized diagnostic work-up.

| **Patient number** | **Initial assessment** | **Reasons for FN+1 classification at standardized diagnostic work-up** | **Antifungal therapy** | **Final Assessment** | **Outcome** | **IFI Classification**  **(2008 EORTC* criteria)** |
| --- | --- | --- | --- | --- | --- | --- |
| 3 | FN+1 | Single thoracic CT scan: new pulmonary infiltrate other than those listed as “major criteria” by the 2008 EORTC* criteria | YES | FUO | Survived | NO IFI |
| 4 | FN+1 | Single thoracic CT scan: new pulmonary infiltrate other than those listed as “major criteria” by the 2008 EORTC* criteria | YES | FUO | Survived | NO IFI |
| 7 | FN+1 | Single thoracic CT scan: new pulmonary infiltrate other than those listed as “major criteria” by the 2008 EORTC* criteria | YES | FUO | Survived | NO IFI |
| 13 | FN+1 | One positive serum galattomannan test | YES | FUO | Survived | NO IFI |
| 17 | FN+1 | Thoracic CT scan: new pulmonary infiltrate other than those listed as “major criteria” by the 2008 EORTC* criteria | NO§ | Bacterial infection | Survived | NO IFI |
| 19 | FN+1 | Nasal sinuses CT scan: early-stage sinusitis other than those listed as “major criteria” by the 2008 EORTC* criteria  Abdominal echography(not included in standardized work-up): atypical splenic lesions | YES | FUO | Survived | NO IFI |
| 22 | FN+1 | Thoracic CT scan: new pulmonary infiltrate other than those listed as “major criteria” by the 2008 EORTC* criteria | NO § | Bacterial infection | Survived | NO IFI |
| 48 | FN+1 | Thoracic CT scan: new pulmonary infiltrate other than those listed as “major criteria” by the 2008 EORTC* criteria | YES | Possible IFI | Survived | Possible IFI **^** |
| 138 | FN+1 | Thoracic CT scan: new pulmonary infiltrate other than those listed as “major criteria” by the 2008 EORTC* criteria | YES | Suspected bacterial infection (Shock) | Survived | NO IFI |
| 157 | FN+1 | Thoracic CT scan: new pulmonary infiltrate other than those listed as “major criteria” by the 2008 EORTC* criteria | YES | FUO | Survived | NO IFI |

**Table S5** Comparison between empiric and preemptive groups according to antifungal prophylaxis (per-protocol)

|  | **EMPIRIC** | |  | **PREEMPTIVE** | |  |
| --- | --- | --- | --- | --- | --- | --- |
| **CHARACTERISTICS** | **POSA***  **Nr. Patients**  **23** | **OTHERS**  **Nr. Patients 17** | **p** | **POSA***  **Nr. Patients**  **28** | **OTHERS**  **Nr. Patients**  **24** | **p** |
| **Antifungal treatment**  -For proven, probable and possible IFI | **23/23 (100%)**  2/23 (9%) | **17/17 (100%)**  1/17 (6%) | **N.S.**  **0.6** | **15/28 (53%)**  9/28 (32%) | **15/24 (62%)**  9/24 (37%) | **0.7**  **0.9** |
| **Final Fungal Infections**  **(2008 EORTC revised criteria)**  *Proven*  *Probable*  *Possible* | **2/23 (9%)**  0  1  1 | **1/17 (6%)**  0  0  1 | **0.6** | **9/28 (32%)**  1  2  6 | **9/24 (37%)**  1  5  3 | **0.1** |
| **Site of Fungal Infections**  CVC/Bacteremia  Lung | -  1 (CT^§^+GM°) | -  - |  | 1 (*G.capitatus*)  2 (CT^§^+GM°) (7%) | 1 (Candidemia)  5 (CT^§^+GM°) (20%) |  |
| **Use of antifungal therapy in**  **patients without proven, probable, and possible IFI** | **21/23 (91%)** | **16/17 (94%)** | **0.7** | **6/28 (21%)** | **6/24 (25%)** | **0.9** |
| **Overall mortality**  **(per protocol)** | **3/23 (12%)** | **4/17 (23%)** | **0.3** | **2/28 (7%)** | **1/24 (4%)** | **0.5** |

***** POSA: posaconazole prophylaxis

^§^ CT: positive CT scan

° GM: positive galattomannan test (a single value >0.5 will be considered positive. Two consecutive samples (in two different days) equal to 0.5 are required for positivity)

**Annex 1: Study protocol**

Annex 1: Flow chart

Annex 2: Definition of Standardized work-up

Annex 3: Definition of patients FN+1

Annex 4: GIMEMA Centres which already agree with the study

**Annex 2:**

**Costs**

1. **Direct costs (Study Protocol: 5.5)**

An exploratory assessment of resource utilization was conducted in both arms over the study period using National Health Service cost of drugs (assessed for a patient weighting 70 Kg) and diagnostic tools, estimated at February 2020 prices.

Data were tabulated according to treatment group and were summarized by appropriate descriptive statistics.

| **Diagnostics** | **Price per Unit (€)** |
| --- | --- |
| Thorax CT scan | 124 |
| Abdominal ultrasonography | 60 |
| Galattomann test | 12 |
| Nasal swab | 7.50 |
| Maxillary CT scan | 120 |

| **Drugs** | **Price per Unit (€)** | **Price "ex factory"(€)** | **Formulation** | **Dose per Unit** | **Daily cost/70Kg (€)** |
| --- | --- | --- | --- | --- | --- |
| ABELCET | 142 | 71 | VL | 100 MG | 247 |
| AMBISOME | 326 | 163 | VL | 50 MG | 652 |
| CASPOFUNGIN | 488 | 244 | VL | 50 MG | 244 |
| VORICONAZOLE | 124 | 62 | VL | 200 MG | 124 |
| VORICONAZOLE | 33 | 17 | TB | 200 MG | 66 |
